# Anomalous origin and aneurysm of the right coronary artery associated with congenital bicuspid aortic valve: MDCT findings

**DOI:** 10.1186/s40064-015-1214-1

**Published:** 2015-08-19

**Authors:** Soobuem Cho, Kyung Nyeo Jeon, Kyungsoo Bae

**Affiliations:** Department of Radiology, Gyeongsang National University School of Medicine and Gyeongsang National University Hospital, 79 Gangnam-ro, Jinju, 660-702 Korea; Department of Radiology, Gyeongsang National University Changwon Hospital, Changwon, Korea

**Keywords:** Coronary artery anomaly, Coronary artery aneurysm, Bicuspid aortic valve, Kawasaki disease, MDCT

## Abstract

Anomalous origin of the coronary artery taking an interarterial course can cause myocardial infarction or sudden death. Association of anomalous origin of the coronary artery with congenital bicuspid aortic valve is rare, and only a few cases have been reported with imaging findings. Coronary artery aneurysms found in young adults are usually non-atherosclerotic. We report MDCT findings of anomalous origin and aneurysm of the right coronary artery associated with congenital bicuspid aortic valve in a 33-year-old man with a history of Kawasaki disease in the childhood, and the key role of MDCT in exact diagnosis and successful management of the complicated disease.

## Background

Anomalous origin of the coronary artery from the wrong sinus of Valsalva and its course passing between the two great arteries has been considered potentially malignant. The association of anomalous origin of the coronary artery and bicuspid aortic valve is rare, and only a few cases with imaging findings have been reported in the literature (Aoyagi et al. 1991; Ayusawa et al. 2010). Coronary artery aneurysm that develops as a complication of Kawasaki disease in infants can remain silent until adulthood. We report multidetector computed tomography (MDCT) findings of anomalous origin and aneurysm of the right coronary artery (RCA) associated with congenital bicuspid aortic valve in a young man who had a history of Kawasaki disease in childhood.

## Case report

A 33-year-old man underwent CT coronary angiography for the evaluation of coronary artery status. He was referred from another hospital where he had received low dose chest CT for health screening and was informed that coronary artery calcification was detected on CT. He did not complain of acute chest pain at this visit but he recalled having intermittent vague chest discomfort. He did not have any cardiovascular risk factors, but had a history of acute febrile illness at 2 years of age that was diagnosed as Kawasaki disease. Electrocardiography findings were nonspecific. MDCT was performed with a 128-slice dual-source CT system (Somatom Definition Flash; Siemens Healthcare, Forchheim, Germany) using the following parameters: detector collimation, 0.6 mm; slice acquisition, 2 × 128 × 0.6 mm; pitch, 0.25; 300 mAs; 100 kVp; gantry rotation time, 280 ms; temporal resolution, 75 ms. Intravenous nonionic contrast material [iomeprol, 400 mg/mL (Iomeron, Bracco)] was administered at a rate of 4–5 mL/s (total, 70 mL) followed by 30 mL of saline at a rate of 3 mL/s. The patient received 0.6 mg of nitroglycerin sublingually just before CT scanning and a beta-blocker was not administered. The effective radiation dose used in CT scanning was 4.9 mSv.

**Fig. 1:**
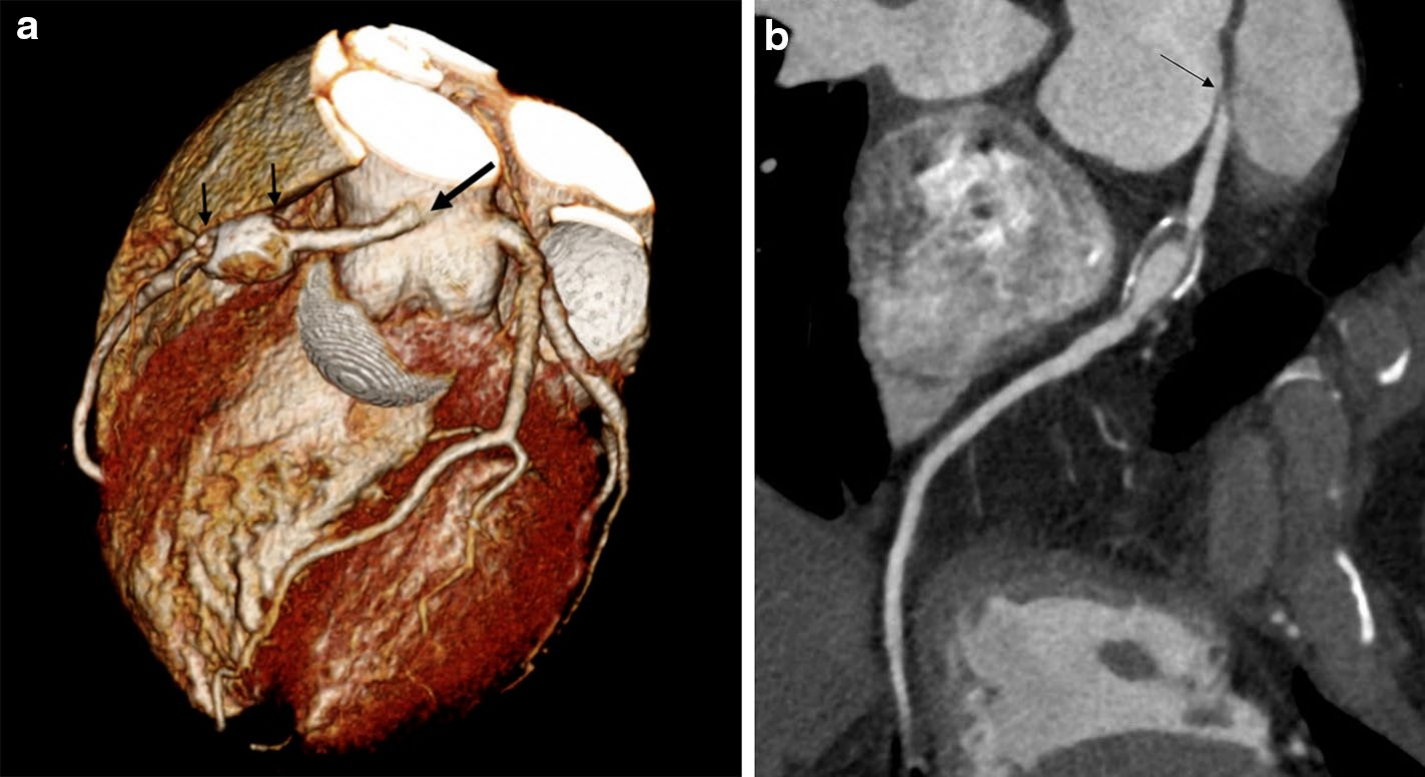
**a** The volume rendering image shows the right coronary artery (RCA) arising from the sinotubular junction just in between the left and right coronary sinus of Valsalva (*arrow*) and courses anteriorly between the aortic root and the pulmonary artery into the right atrioventricular groove. There is a saccular aneurysm (*small arrows*) in the proximal portion of the RCA. **b** Curved multiplanar reformatted image of the RCA shows an acute take-off angle of the origin (*arrow*) and ring calcification and narrowing of the aneurysmal segment caused by a mural thrombus.

**Fig. 2:**
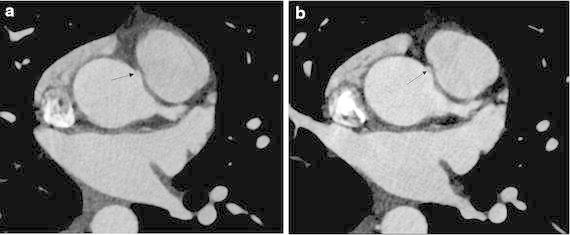
**a** Axial image taken at the level of the right coronary artery (RCA) shows a nearly tangential origin of the RCA and a narrow RCA orifice (*arrow*). **b** In the systolic phase, narrowing of the RCA orifice is accentuated (*arrow*).

**Fig. 3:**
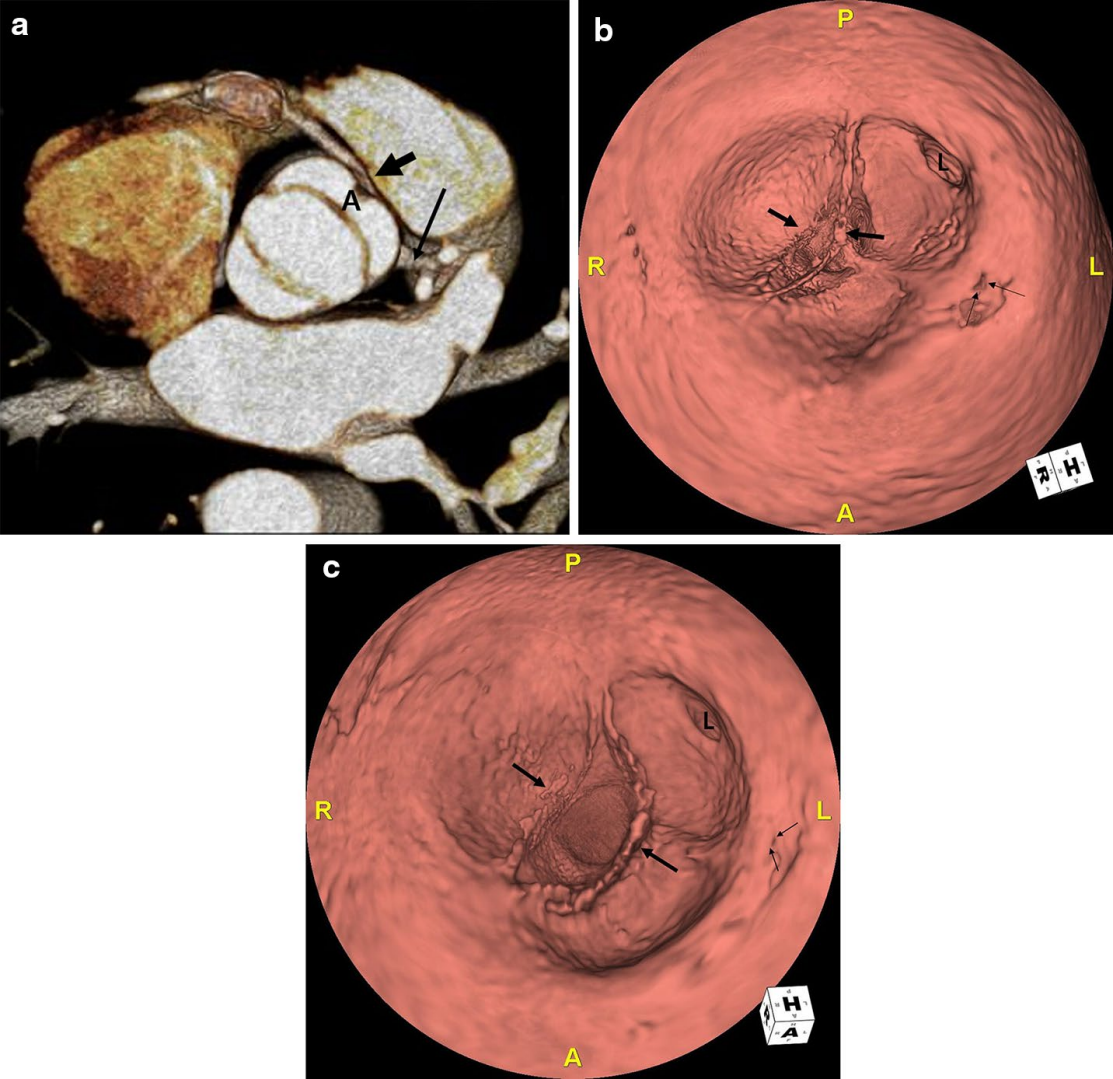
**a** 3D CT image in systole shows bicuspid aortic valve with a symmetric and elliptical orifice. Note the right (*arrow*) and left (*long arrow*) coronary arteries originating from anterior cusp (A) formed by fusion of the right and left coronary cusp. **b** Three-dimensional virtual angioscopic image of the aortic root shows the bicuspid aortic valve (*arrows*), ostium of the left main coronary artery (L), and a slit-like orifice of the right coronary artery (*thin arrows*) which is located high at the sinotubular junction. **c** In the systolic phase, the orifice of the RCA is nearly effaced (*thin arrows*).

The volume rendering image of the heart revealed that the RCA arose from the sinotubular junction in between the left and right coronary sinus of Valsalva and coursed anteriorly between the aortic root and the pulmonary artery into the right atrioventricular groove (Fig. 1a). A saccular aneurysm in the proximal portion of the RCA was noted. Curved multiplanar reformation image of the RCA showed an acute take-off angle of the RCA origin and focal significant narrowing at the proximal portion of the aneurysm (Fig. 1b). Axial images at the level of RCA origin showed further narrowing of the RCA orifice at the systolic phase (Fig. 2), but the anomalous coronary artery did not have an intramural course within the aortic wall. Bicuspid aortic valve had two completely developed cusps without raphe (Fig. 3a). Three-dimensional virtual angioscopic image of the aortic root showed a bicuspid aortic valve and normal ostium of the left main coronary artery. A slit-like orifice of the right coronary artery was located high at the sinotubular junction between the right and left coronary sinus (Fig. 3b) and was nearly effaced in the systolic phase (Fig. 3c). Echocardiography revealed bicuspid aortic valve with mild aortic regurgitation and normal left ventricular function. One month later, the patient underwent bypass graft surgery for the RCA with good recovery.

## Discussion

Coronary anomalies are detected in about 1 % of the general population by coronary angiography and have little clinical significance (Angelini et al. 2002). However, a minority of coronary artery anomalies, particularly in which the coronary artery takes an interarterial course, are known to have a risk of myocardial ischemia or sudden cardiac death (Rigatelli et al. 2005). Several possible causes of myocardial ischemia in cases with anomalous coronary artery origin from the wrong aortic sinus with a course between the aorta and the pulmonary artery have been suggested: acute angle take-off of the coronary artery producing a slit-like lumen; closure of the abnormal coronary orifice by a valve-like ridge at aortic expansion; compression of the artery when it courses within the aortic wall (intramural course) or between the aorta and the pulmonary artery; and spasm of the anomalous coronary artery (Basso et al. 2000; Virmani et al. 1984). Virmani et al. (1984), after observing postmortem coronary arteries in victims of sudden death, postulated that the presence of ostial valve-like ridges with acute angle take-offs in coronary arteries cause ostial compression with aortic dilatation. By acting as an occlusive valve, the ostial valve-like ridges in acute take-offs may cause coronary spasm and sudden death. In the present case, MDCT showed narrowing of the RCA origin with an acute take-off angle from the aorta, and ostial narrowing was accentuated in the systolic phase although we could not confirm the presence of an ostial valve-like ridge.

Our patient also had an aneurysm with mural thrombus and ring calcification in the proximal portion of the anomalous RCA, which can be attributed to Kawasaki disease. In Kawasaki disease, coronary artery aneurysms may develop as a late complication and remain silent until adolescence or young adulthood when myocardial infarction or sudden death occurs (Okura et al. 2013). Burns et al. (1996) performed a retrospective survey of 74 published cases of coronary artery disease attributed to antecedent Kawasaki disease in young adults, and stated that history of Kawasaki disease should be sought in all young adults who present with acute myocardial infarction or sudden death. Vasculitis of the coronary vasa vasorum leads to endothelial damage and coronary artery aneurysm formation, and slow flow within the aneurysm predisposes to thrombus formation or atherosclerotic change (Okura et al. 2013; Burns et al. 1996). Moreover, in our patient, the narrow orifice in the presence of anomalous origin could also have produced hemodynamic alteration and further endothelial injury, contributing to acceleration of aneurysm formation and thrombosis in the RCA (De-Giorgio et al. 2011).

A bicuspid aortic valve is one of the most frequent congenital cardiac malformations in adults and it may be isolated or associated with other congenital heart diseases. There is increased variation in the coronary anatomy in patients with bicuspid aortic valves (Fedak et al. 2002). The association of anomalous origin of coronary arteries and congenital bicuspid aortic valve has only been sporadically reported in the literature (Aoyagi et al. 1991; Ayusawa et al. 2010). According to the observation of embryonic heart development, differentiation of aortic valve is completed during the similar period in which the proximal coronary arteries develop as endothelial outgrowth from the aortic sinus (Hutchins et al. 1988). Thus, it could be postulated that a common developmental defect produced both anomalies in this patient. Some cardiac surgeons support the routine use of preoperative coronary angiography to assess the status of coronary arteries before aortic valve operation (Barriales-Villa et al. 2003). However, as seen in our case, technically advanced MDCT can replace coronary angiography. Cardiac MDCT can provide comprehensive anatomic information about this complicated anomaly through three-dimensional reconstruction, and also functional information of bicuspid aortic valve. In addition, aortic dilatation, which frequently occurs as a complication of the bicuspid aortic valve, can be evaluated by MDCT.

## Conclusion

To the best of our knowledge, this is the first reported case of congenital bicuspid aortic valve associated with an anomalous origin of the RCA, complicated by late sequelae of Kawasaki disease and diagnosed with MDCT. MDCT is an effective and useful tool for exact assessment of coronary artery status and associated anomalies, resulting in timely management.

## Consent for publication

Informed consent for publication of this report and any accompanying images was obtained from the patient.

## Abbreviations

MDCT: multidetector computed tomography
RCA: right coronary artery
CT: computed tomography
